# College and Hospital Notes

**Published:** 1900-09

**Authors:** 


### COLLEGE AND HOSPITAL NOTES.

THE medical department of the University of Buffalo will open its fifty-fifth regular session on Monday evening, September 24, 1900, when an inaugural address will be delivered. The following day, Tuesday, the lecture course will commence and continue for the next thirty weeks, during which there will be instruction by lectures, recitations, laboratory work and clinics. Full particulars and a catalogue may be obtained by addressing Professor John Parmenter, M. D., secretary of the University, at the college building, 24 High Street, Buffalo, N.Y.

---

THE Albany Medical College Alumni Association offers the Schuyler alumni prize for 1901, established by Dr. Clarkson C. Schuyler, A. M. C., '75. It is a yearly prize of $100 to be awarded at the annual meeting of the Alumni Association, for the best essay written by a graduate of this college on some prescribed subject. The subject for next year is: "The Influence of the Discovery of the Relation of Bacteria to Disease on the Practice of Medicine Exclusive of Surgery."

Competing essays, signed by some assumed name or motto, and accompanied by a sealed envelope superscribed in the same manner and containing within the author's real name and address, are to be sent to Dr. Samuel B. Ward, Albany, of the prize committee, on or before March 1, 1901.

